# The Methanolic Extract from* Murraya koenigii* L. Inhibits Glutamate-Induced Pain and Involves ATP-Sensitive K^+^ Channel as Antinociceptive Mechanism

**DOI:** 10.1155/2016/3790860

**Published:** 2016-10-12

**Authors:** Nushrat Sharmin Ani, Sudip Chakraborty, Md. Moniruzzaman

**Affiliations:** Department of Pharmacy, Stamford University Bangladesh, 51 Shiddheswari Road, Dhaka 1217, Bangladesh

## Abstract

*Murraya koenigii* L. is a perennial shrub, belonging to the family Rutaceae. Traditionally, the leaves of this plant are extensively used in treatment of a wide range of diseases and disorders including pain and inflammation. Although researchers have revealed the antinociceptive effects of this plant's leaves during past few years, the mechanisms underlying these effects are still unknown. Therefore, the present study evaluated some antinociceptive mechanisms of the methanolic extract of* M. koenigii* (MEMK) leaves along with its antinociceptive potential using several animal models. The antinociceptive effects of MEMK were evaluated using formalin-induced licking and acetic acid-induced writhing tests at the doses of 50, 100, and 200 mg/kg. In addition, we also justified the possible participations of glutamatergic system and ATP-sensitive potassium channels in the observed activities. Our results demonstrated that MEMK significantly (*p* < 0.01) inhibited the pain thresholds induced by formalin and acetic acid in a dose-dependent manner. MEMK also significantly (*p* < 0.01) suppressed glutamate-induced pain. Moreover, pretreatment with glibenclamide (an ATP-sensitive potassium channel blocker) at 10 mg/kg significantly (*p* < 0.05) reversed the MEMK-mediated antinociception. These revealed that MEMK might have the potential to interact with glutamatergic system and the ATP-sensitive potassium channels to exhibit its antinociceptive activities. Therefore, our results strongly support the antinociceptive effects of* M. koenigii* leaves and provide scientific basis of their analgesic uses in the traditional medicine.

## 1. Introduction

*Murraya koenigii* L. Sprengel (family: Rutaceae) is a small and strong smelling perennial shrub that commonly grows in South East Asian countries and known as curry leaf plant. This plant is widely cultivated for its leaves which possess characteristic flavor and aroma and are used as a condiment and flavoring agent in ethnic foods [1, 2]. Traditionally, the leaves of this plant are used to treat a wide range of diseases and disorders such as pain, inflammation, itching, cancer, diabetes, and blood disorders [1, 3]. Previous phytochemical screening revealed a rich profile of simple phenolic acids including gallic, cinnamic, tannic, caffeic, ferulic, chlorogenic, and vanillic acids in curry leaves [1]. The essential oils identified in these leaves include *α*-pinene, *β*-phellandrene, (E)-caryophyllene, *α*-selinene, tetradecanoic acid, hexadecanoic acid, c-eudesmol, a-muurolol, (Z,E)-farnesol, and (Z,Z)-farnesol [4]. The leaves also contain monoterpene derived hydrocarbons and alcohols possessing antioxidant potentials* in vitro* [5]. In 2013, the research groups of Nakamura and Ma isolated six new carbazole alkaloids including karapinchamines A and B [6], N-benzyl carbazole-A, N-benzyl carbazole-B, iso-koenidine, and iso-koenigine along with fourteen other known carbazole alkaloids having hepatoprotective and anticancer properties [7].

Along with this rich phytochemical profile, researchers also revealed the hypolipidemic, antidiabetic [1], hepatoprotective, antidiarrheal, and antioxidant properties [3] of curry leaves. Although their antinociceptive activity is well described [8, 9], the mechanisms of actions are not revealed yet. Therefore, for the first time, we evaluated the possible participations of glutamatergic system as well as ATP-sensitive K^+^ channels in methanolic extract of* M. koenigii*- (MEMK-) mediated antinociception in mice. In addition, we also justified the action of MEMK in two conventional pain models including acetic acid-induced writhing and formalin-induced licking tests.

## 2. Materials and Methods

### 2.1. Plant Material and Extraction

*M. koenigii* leaves were collected from Baily Road, Dhaka, Bangladesh, in April 2015. The samples were then identified by Busra Khan, Principle Scientific Officer, Bangladesh National Herbarium, with a voucher number of DACB: 41516. Powdered dried leaves (300 g) were macerated with 500 mL of methanol with occasional stirring for 72 hours at 25 ± 2°C temperature. Then the collected filtrate was dried using rotary evaporator and normal air flow, respectively, resulting in 16.50 g of extract (yield: 5.50%). This crude extract was further used for the acute toxicity and antinociceptive activity analysis.

### 2.2. Drugs and Reagents

Diclofenac sodium and glibenclamide were purchased from Square Pharmaceuticals Ltd. (Dhaka, Bangladesh). Methanol, 99% dimethylsulfoxide (DMSO), l-glutamic acid, acetic acid, and formalin were procured from Merck (Darmstadt, Germany).

### 2.3. Phytochemical Screening

The crude methanolic extract of* M. koenigii* was qualitatively tested to detect the presence of alkaloids, glycosides, tannins, carbohydrates, reducing sugars, flavonoids, and saponins according to the standard protocols [10].

### 2.4. Animals

Male Swiss Albino mice of 20–25 g body weight were collected from the Animal Research Branch of the International Center for Diarrheal Disease and Research, Bangladesh (ICDDR,B). Animals were maintained under standard laboratory conditions (maintaining 12 h light/dark cycle, 25 ± 2°C room temperature, and 55–65% relative humidity) with ICDDR,B formulated standard diet and clean water provided* ad libitum*. The animals were kept to the laboratory environment for a period of 14 days before the experiments and fasted overnight prior to the experiments. All experimental animals were treated following the Ethical Principles and Guidelines for Scientific Experiments on Animals (1995) formulated by The Swiss Academy of Medical Sciences and the Swiss Academy of Sciences. All protocols conducted in this study were approved by the Institutional Ethics Committee of Stamford University Bangladesh (SUB/IAEC/15.07).

### 2.5. Drugs and Treatments

Diclofenac sodium (10 mg/kg) was administered intraperitoneally (i.p.) 15 min before the nociceptive stimuli. In all experiments, DMSO (vehicle, 0.1 mL/mouse) or MEMK (50, 100, and 200 mg/kg) were administered orally 30 min prior to the induction of nociception. Moreover, glibenclamide at the dose of 10 mg/kg was given 15 min prior to the standard drug in addition to MEMK to justify the possible involvement of K^+^ channel.

### 2.6. Acute Toxicity Test

The animals were divided into desired groups, with each group containing five to seven animals. MEMK was administered to the animals at the doses of 1000, 2000, and 3000 mg/kg p.o. and animals were observed for any allergic reaction or mortality for next 72 h. In the meantime, they were allowed free access of food and water* ad libitum* [11].

### 2.7. Formalin-Induced Nociception

The procedure used was similar to that previously described by Santos and Calixto [12]. Animals were randomly selected for each group and pretreated with standard drug or MEMK. 60 min after MEMK and 30 min after drug, 20 *μ*L of 2.5% formalin was injected in the ventral surface of the right hind paw of the mice. Then the animals were observed and numbers of lickings and bitings of the injected paw were recorded for 0–5th min (neurogenic or early phase) and 15–30th min period (inflammatory or late phase) after formalin injection.

### 2.8. Acetic Acid-Induced Writhing Test

Nociception in mice was induced with intraperitoneal injection of 0.6% acetic acid (10 mL/kg) 15 min after the drug and 30 min after MEMK treatments. 5 min after the acetic acid injection, mice were observed and the total number of writhings (abdominal constriction and stretching of hind limb) was counted for 30 min [13].

### 2.9. Glutamate-Induced Nociception

In glutamate test, mice were treated with extract or standard drug as mentioned above. 30 min after extract and 15 min after drug, 20 *μ*L of glutamate (10 *μ*M/paw) was injected in the subplantar region of the right hind paw of mice. Mice were then observed for 15 min and numbers of lickings and bitings of the injected paw were counted as a score of nociception [14].

### 2.10. Involvement of ATP-Sensitive K^+^ Channel Pathway

The possible involvement of ATP-sensitive K^+^ channel in MEMK-mediated antinociceptive effect was evaluated using previously described methods by Mohamad et al. and Perimal et al. [15, 16]. 15 min before the treatment with MEMK (200 mg/kg), animals were treated with glibenclamide (10 mg/kg). Following 30 min of MEMK administration, animals were challenged with i.p. injection of 0.6% acetic acid and immediately placed in a chamber. Then the number of writhings was recorded for 30 min, starting from 5 min after injection.

### 2.11. Statistical Analysis

The results are expressed as mean ± SEM. The statistical analysis was performed using one-way analysis of variance (ANOVA) followed by Dunnett's* post hoc* test using SPSS software. ED_50_ values were calculated using GraphPad Prism and figures were drawn using SigmaPlot software.

## 3. Results and Discussion

The present study evaluated the actions of MEMK on different animal models of pain such as formalin-induced licking and acetic acid-induced writhing tests. This study also revealed the probable underlying mechanisms including involvement of glutamatergic system as well as ATP-sensitive K^+^ channels in the antinociceptive activities of MEMK. Moreover, our study demonstrated that oral administration of MEMK at 1000–3000 mg/kg doses did not produce any allergic reactions, abnormal behavior, and mortality of the animals within 72 h of observation period, revealing its nontoxic profile within our experimental doses tested up to 3000 mg/kg.

**Figure 1:**
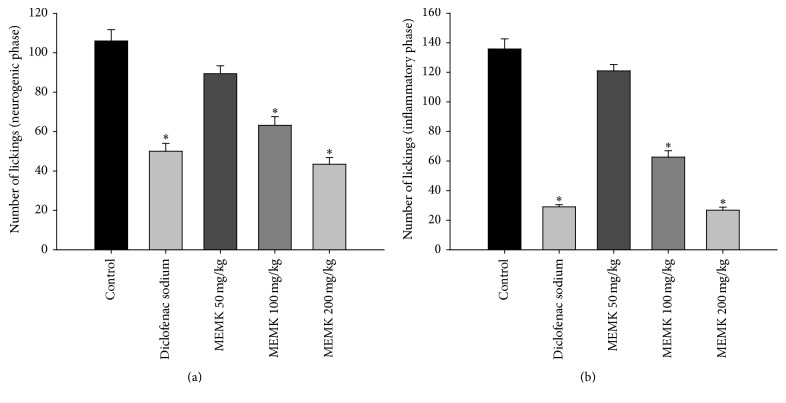
Response frequencies of the right hind paws injected with 2.5% formalin in early phase (a) and in late phase (b) of formalin test. Each group represents the mean ± SEM (*n* = 5–7). Statistical analysis was performed using one-way ANOVA followed by Dunnett's* post hoc* test. ^*∗*^ *p* < 0.01 compared to control.

**Figure 2:**
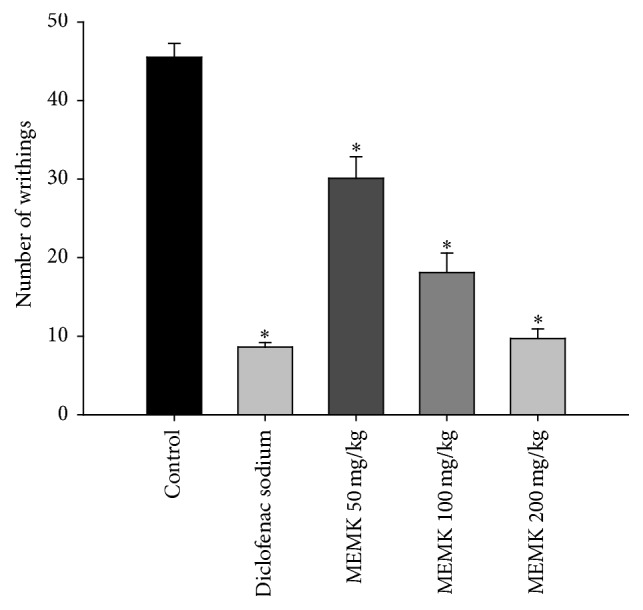
Effects of MEMK in the nociception induced by 0.6% acetic acid in mice. Each group represents the mean ± SEM (*n* = 5–7). Statistical analysis was performed using one-way ANOVA followed by Dunnett's* post hoc* test. ^*∗*^ *p* < 0.01 compared to control.

It is well established that the neurogenic pain in first phase of formalin test is mediated through the activation of substance P and bradykinins. On the other hand, several amines including histamine and serotonin, bradykinins, and prostaglandins were reported to play a critical role in the late phase to produce inflammatory pain [12]. Our results demonstrated that the acute administration of MEMK significantly (*p* < 0.01) suppressed the licking behavior of the animals in both phases of formalin test. The percentages of licking inhibitions were calculated as 15.66, 40.38, and 59.06 in first phase and 10.90, 53.90, and 80.27 in late phase for the doses of 50, 100, and 200 mg/kg, respectively (Figures 1(a) and 1(b)). ED_50_ values were also calculated as 146.40 mg/kg for first phase and 100.70 mg/kg for the late phases. A previously published work reported by Gupta et al. [17] supports these findings, where they found that the aqueous extract of* M. koenigii* at the doses of 400 and 800 mg/kg inhibited formalin-induced pain in mice. In addition, the intraperitoneal injection of acetic acid causes pH imbalance and the activation of peritoneal resident cells (macrophages and mast cells). This influences an elevated release of several inflammatory mediators such as prostaglandin E2 and TNF-*α* [13]. As depicted in Figure 2, the results demonstrated that all doses of MEMK significantly (*p* < 0.01) inhibited acetic acid-induced writhing with ED_50_ value of 77.85 mg/kg. The percentages of writhing inhibitions were calculated as 33.85, 60.22, and 78.68 for 50, 100, and 200 mg/kg doses, respectively. Moreover, the petroleum ether extract and the alkaloid fraction of* M. koenigii* leaves were also previously reported to have writhing inhibitory properties in animals [9, 18]. Therefore, our findings and previously reported works strongly support the fact that MEMK is endowed with formalin-induced and acetic acid-induced pain inhibitory properties.

**Figure 3:**
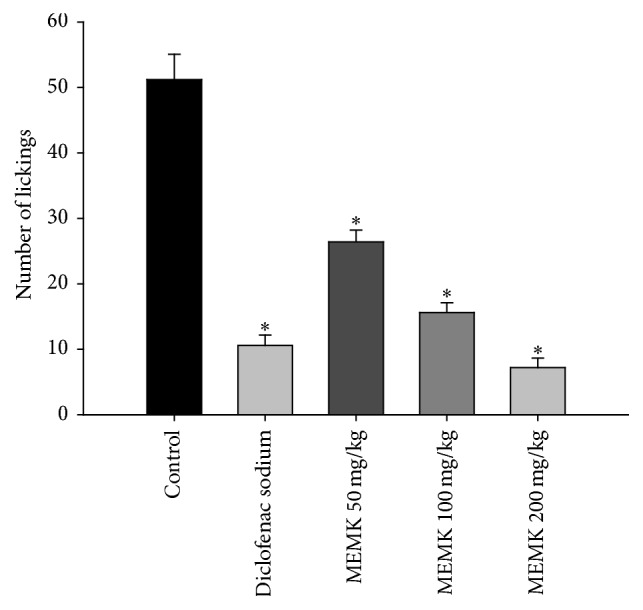
Effects of MEMK on glutamate-induced nociception in mice. Each group represents the mean ± SEM (*n* = 5–7). Statistical analysis was performed using one-way ANOVA followed by Dunnett's* post hoc* test. ^*∗*^ *p* < 0.01 compared to control.

**Figure 4:**
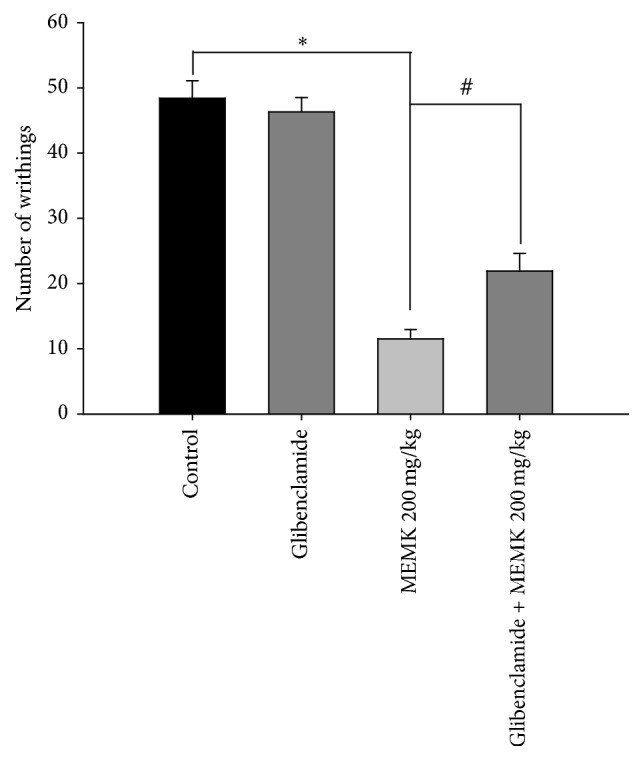
Reversal effects of glibenclamide in MEMK-mediated antinociception. Each group represents the mean ± SEM (*n* = 5–7). Statistical analysis was performed using one-way ANOVA followed by Dunnett's* post hoc* test. ^*∗*^ *p* < 0.01 compared to control and ^#^ *p* < 0.05 compared to MEMK 200 mg/kg.

In order to elucidate the antinociceptive mechanism of action of MEMK, the nociception induced by intraplantar injection of glutamate was tested. Glutamate is an excitatory amino acid widely known to play major role in pain perceptions by acting through peripheral, spinal, and supraspinal sites of actions using both N-methyl-D-aspartate (NMDA) and non-NMDA receptors [14]. Additionally, glutamate is also reported to induce the synthesis and release of several proinflammatory mediators including nitric oxide (NO) and NO-related and arachidonic acid-related substances in both central and peripheral nervous systems [19]. We found that MEMK significantly (*p* < 0.01) inhibited the noxious stimuli induced by glutamate in a dose-dependent manner. The estimated percentages of inhibitions were 48.44, 69.53, and 85.94 for 50, 100, and 200 mg/kg doses, respectively (ED_50_: 52.76 mg/kg). Besides, the reference drug diclofenac sodium also significantly (*p* < 0.01) reduced the pain behavior of the animals accounting for 79.30% of licking inhibition (Figure 3). Considering the above findings, it is conceivable that the components of MEMK might have the potential to interact with glutamatergic system to demonstrate its antinociceptive activity. Moreover, it was also found that administration of glibenclamide (an ATP-sensitive potassium channel antagonist) at 10 mg/kg alone did not alter the writhing episodes evoked by the injection of 0.6% acetic acid. However, given together with the extract, glibenclamide significantly (*p* < 0.05) reversed the antinociceptive effects of MEMK (Figure 4). Substantial scientific reports revealed that glibenclamide specifically blocks only the ATP-sensitive K^+^ channels but does not affect other types like Ca^2+^ activated and voltage-gated K^+^ channels [15]. Therefore, the results might suggest that MEMK involves the opening of ATP-sensitive potassium channels which allows the efflux of K^+^ ion, leading to repolarization or hyperpolarization of the membrane, which reduces the membrane excitability [16].

Our preliminary phytochemical analysis revealed the presence of alkaloids, glycosides, tannins, carbohydrates, reducing sugars, and flavonoids in MEMK (Table 1). It has been reported that the carbazole alkaloids as well as the flavonoids isolated from curry leaves possess potent antioxidant properties [1, 20, 21]. Moreover, the chemical compounds with antioxidant properties are generally known to relieve pain through the prevention of lipid peroxidation as well as the synthesis and release of different inflammatory mediators [22]. Therefore, it is conceivable that the phytochemicals present in the extract participate at least in a part of the observed activities of MEMK.

## 4. Conclusion

In conclusion, the results of the present study demonstrated the antinociceptive actions of the methanolic extract of* M. koenigii* leaves and provided the scientific evidence regarding their traditional uses as an analgesic agent. This study also uncovered the probable participation of glutamatergic system as well as ATP-sensitive potassium channels in MEMK-mediated antinociception.

## Figures and Tables

**Table 1:** Phytochemicals identified in MEMK.

Phytochemicals	Names of the tests	Expected changes	Results
Alkaloids	Mayer's test	Yellowish buff color precipitate	+
	Hager's test	Yellow crystalline precipitate	+
	Wagner's test	Brown or deep brown precipitate	−
	Dragendorff's test	Orange or orange-brown precipitate	+
	Tannic acid test	Buff color precipitate	−

Tannins	Ferric chloride test	Blue green color	+
	Alkaline reagent test	Yellow to red precipitate	+

Glycosides	General test	Yellow color	+
	Test for glucoside	Production of brick-red precipitation	+

Carbohydrates	Molisch's test	A red or reddish violet ring is formed at the junction of two layers, and on shaking a dark purple solution is formed	−
	Barfoed's test (general test for monosaccharides)	Red precipitate	+
	Fehling's test	A red or brick-red precipitate	+
	Test for reducing sugar	A brick-red precipitate	+

Flavonoids	Hydrochloric acid reduction test	Red color	+

Saponins	Frothing test	Formation of stable foam	−
